# Posaconazole Alone and in Combination with Caspofungin for Treatment of Experimental *Exserohilum rostratum* Meningoencephalitis: Developing New Strategies for Treatment of Phaeohyphomycosis of the Central Nervous System

**DOI:** 10.3390/jof6010033

**Published:** 2020-03-05

**Authors:** Ruta Petraitiene, Vidmantas Petraitis, BoBo Win Maung, Ethan Naing, Povilas Kavaliauskas, Thomas J. Walsh

**Affiliations:** 1 Transplantation-Oncology Infectious Diseases Program, Division of Infectious Diseases, Department of Medicine, Weill Cornell Medicine of Cornell University, New York, NY 10065, USA; vip2007@med.cornell.edu (V.P.); bobowinmg@gmail.com (B.W.M.); etn2001@med.cornell.edu (E.N.); pok4001@med.cornell.edu (P.K.); thw2003@med.cornell.edu (T.J.W.); 2 Institute of Infectious Diseases and Pathogenic Microbiology, LT-59115 Prienai, Lithuania; 3 Department of Pediatrics, Weill Cornell Medicine of Cornell University, New York, NY 10065, USA; 4 Department of Microbiology & Immunology, Weill Cornell Medicine of Cornell University, New York, NY 10065, USA

**Keywords:** experimental *Exserohilum rostratum* meningoencephalitis, posaconazole and caspofungin combination, rabbits

## Abstract

Phaeohyphomycosis of the central nervous system (CNS) is a life-threatening infection associated with severe morbidity. New approaches to treatment of CNS phaeohyphomycosis are critically needed. We therefore studied posaconazole with or without caspofungin for treatment of experimental CNS phaeohyphomycosis caused by *Exserohilum rostratum*. Each clinical isolate of *E. rostratum* isolate was inoculated intracisternally with 1.0 × 10^6^ microconidia to fully anesthetized New Zealand White rabbits. Profound persistent neutropenia and immunosuppression were established and maintained using cytarabine and methylprednisolone, respectively. Study groups consisted of posaconazole suspension administered as oral formulation at 10 (PSC10) or 20 (PSC20) mg/kg, caspofungin (CFG) at 2 mg/kg intravenously (IV), combinations of PSC10+CFG or PSC20+CFG, and untreated controls (UC). Posaconazole produced a significant reduction of residual fungal burden of *E. rostratum* in cerebrum, cerebellum, spinal cord, and paravertebral muscle (*p* < 0.01), in comparison to UC. The combination of PSC10+CFG and PSC20+CFG achieved full clearance of residual fungal burden from cerebrum, while only PSC20+CFG treated rabbits demonstrated clearance from cerebellum, spinal cord, and paravertebral muscle (*p* < 0.01). These data correlated with the significant reduction of CSF (1→3)-β-d-glucan levels in rabbits treated with PSC20 and PSC20+CFG in comparison to those of UC (*p* < 0.05). Posaconazole alone or in combination with caspofungin demonstrated significant antifungal efficacy in the treatment of experimental *E. rostratum* meningoencephalitis and warrants further study for treatment of CNS phaeohyphomycosis.

## 1. Introduction

Phaeohyphomycosis of the central nervous system (CNS) is a life-threatening infection associated with severe morbidity and high mortality [1,2,3,4,5,6]. Among the dematiaceous fungi causing CNS phaeohyphomycosis, *Exserohilum rostratum* has been found to be especially virulent as a CNS pathogen following the administration of contaminated methylprednisolone, resulting in meningoradiculitis, brain abscesses, ensuing neurologic deficits, and death [7,8]. 

Although posaconazole is cited as a potential treatment option for the management of CNS phaeohyphomycosis [9], it is not well defined or supported by preclinical in vivo or clinical data for this indication. While voriconazole or liposomal amphotericin B may be effective in some patients, mortality and morbidity remains high and toxicity from either agent may be dose limiting. 

We hypothesized that posaconazole, as an alternative single agent, and its combination with caspofungin may be an effective alternative for the treatment of CNS phaeohyphomycosis. Supporting this hypothesis, posaconazole has in vitro activity against a wide range of dematiaceous molds [9,10,11,12,13] and caspofungin, in combination with mold-active triazoles, has in vitro additive or synergistic activity against *Aspergillus* spp. [14,15,16,17]. In order to test this hypothesis, we therefore studied posaconazole alone and in combination with caspofungin in the rabbit model of *E. rostratum* meningoencephalitis.

## 2. Materials and Methods

### 2.1. Organisms and Inoculation

#### 2.1.1. Organisms

Two isolates of *E. rostratum* (12-2725, 12-2809), that were obtained from patients suffering from *E. rostratum* meningoencephalitis, were used for the study. 

#### 2.1.2. Inoculum Preparation and Inoculation

For preparation of the inoculum, two *E. rostratum* isolates were subcultured from a frozen stock culture stored at −80 °C on potato dextrose agar plates, incubated for 24 h at 37 °C and then kept on subcultured agar plates at room temperature for 7–14 days prior to use. Abundant conidia were elaborated under these conditions. Under a laminar flow hood, the plates were carefully filled with 10 mL of 0.025% Tween-20 saline solution, to form a suspension free of aggregated conidia. A 10-mL pipette was used to gently scrape the conidia from the mycelium and to repeatedly aspirate the suspension. The process was repeated twice for the same plate, and using multiple plates, a suspension of *E. rostratum* was collected into 50-mL conical tubes. The conical tubes were then centrifuged at 2500× *g* for 10 min, the supernatant and floating hyphae removed until 5 mL (conidia and fluid) remained at the bottom of tubes. Suspensions from 50-mL tubes were collected into one, centrifuged and the supernatant removed. Conidia were resuspended with 0.025% Tween-20 to a final volume. The number of *E. rostratum* conidia was counted using a standard hemacytometer. The final concentration was adjusted to a 1.0 × 10^6^ inoculum of microconidia in 500 µL of sterile 0.9% normal saline for each rabbit. Prior to the intracisternal administration of a 1.0 × 10^6^ inoculum, an aliquot of 500 µL of CSF was withdrawn from each fully anesthetized rabbit by inserting a 25 G needle into the cisterna magna. The puncture was performed in the midline halfway between the cranial edges of the wings of the atlas and below external occipital protuberance at the atlantooccipital site. 

### 2.2. Animals

Healthy female New Zealand White rabbits weighing 2.6 to 3.6 kg (Covance Research Products, Inc., Denver, PA) at the time of meningeal inoculation were used in all experiments. All experimental groups were studied simultaneously. All rabbits were monitored under humane care and use of standards in facilities accredited by the Association for Assessment and Accreditation of Laboratory Animal Care International, according to the guidelines of the National Research Council for the care and use of laboratory animals, and under the approval of the Animal Care and Use committee of the Weill Cornell Medicine, New York, NY (2012—0093). Rabbits were individually housed and maintained with water and standard rabbit feed ad libitum. 

### 2.3. Induction and Maintenance of Neutropenia and Immunosuppression

#### 2.3.1. Neutropenia

The experiments started with an initial course of Ara-C at a dosage of 440 mg/m^2^ of for 5 consecutive days (days 1–5) for induction of neutropenia and continued with the administration of Ara-C at dosage of 440 mg/m^2^ on days 8–9, 13–14, and 18–19 of the experiment to maintain profound and persistent neutropenia (< 100/µL). 

#### 2.3.2. Immunosuppression

Simultaneously, methylprednisolone was administered at 5 mg/kg of body weight for immunosuppression on day 1 and continued throughout the experiment, to inhibit macrophage activity and to facilitate establishment of infection.

#### 2.3.3. Antibiotics

Ceftazidime 75 mg/kg twice daily, gentamicin 5 mg/kg every other day, vancomycin 15 mg/kg daily were administered IV on day 4 of the study for the prevention of opportunistic bacterial infections. In addition, all rabbits received 50 mg of vancomycin per liter of drinking water, to prevent antibiotic-associated diarrhea due to *Clostridium spiroforme*.

#### 2.3.4. White Blood Cell Counts

Total leukocyte counts and the percentages of neutrophils were monitored twice weekly. 

The rabbits were inoculated intracisternally on day 6 of the study, with the inoculum of *E. rostratum* at the concentration of 1.0 × 10^6^ of microconidia.

The antifungal treatment was started on day 7 (24 h after inoculation) and continued for 13 days in surviving rabbits.

### 2.4. Inoculum Response Relationship

Initial studies characterizing the tissue burden of *E. rostratum* in the cerebrum, cerebellum, and spinal cord were studied in three intracisternal inoculum groups: 1.0 × 10^2^, 1.0 × 10^4^, and 1.0 × 10^6^ microconidia. There were four rabbits in each inoculum group. The tissue samples were harvested when rabbits were euthanized at the end of the study, or after rabbits were euthanized following humane end points.

### 2.5. Antifungal Compounds and Experimental Groups

Antifungal therapy was initiated on day 7 (24 h after intracisternal inoculation) and continued throughout the course of each experiment for 13 days in surviving rabbits. Posaconazole was administered as an oral formulation at 10 (PSC10) or 20 (PSC20) mg/kg. Caspofungin (CFG) was administered IV at 2 mg/kg/d, in combination with posaconazole as PSC10+CFG or PSC20+CFG. There were six rabbits in each treatment group and untreated control rabbits.

### 2.6. Outcome Variables

Antifungal activity in vivo was determined by the quantitative clearance of *E. rostratum* from tissues and by the resolution of CSF (1→3)-β-d-glucan. 

#### 2.6.1. Quantitative Cultures

Sections of cerebrum (right hemisphere), all cerebellum tissue, upper cervical spinal cord tissue, and muscles from the area around the inoculum injection site (above cisterna magna) were resected, weighed, and homogenized (Stomacher 80; Tekmar Corp., Cincinnati, OH) in sterile reinforced polyethylene bags (Nasco Whirl-Pak, Atlanta, GA) with 2 or 5 mL of sterile 0.9% normal saline for 30 s [18]. Tissue homogenate dilutions (10^−1^ and 10^−2^) were prepared in sterile NS. Aliquots (100 μL) from homogenates and homogenate dilutions were plated onto Sabouraud glucose agar plates and incubated at 37 °C for the first 24 h and then held at room temperature for another 24 h. Data were graphed as the mean of log colony forming units (CFU)/g ± standard error of the mean (SEM).

#### 2.6.2. (1→3)-β-d-Glucan Assay 

A CSF sample from each rabbit was collected on the last day of experiment during euthanasia, for determination of (1→3)-ß-d-glucan levels. The assay was performed according to the manufacturer’s instructions (Fungitell; Associates of Cape Cod, Inc., Falmouth, MA, USA). Briefly, aliquots of 5 μL of plasma or CSF were added to duplicate wells of a 96-well microtiter plate and pretreated for 10 min at 37 °C with an alkaline reagent (20 μL; 0.125 M KOH/0.6 M KCl). An aliquot of 25 μL of the standards (100 to 6.25 pg/mL pure pachyman, a linear β-glucan) was then added to each well. An aliquot of 100 μL of Fungitell reagent (lyophilized β-glucan-specific *Limulus* amebocyte lysates) was reconstituted with 2.8 mL of glucan-free reagent-grade water, followed by 2.8 mL of Pyrosol reconstitution buffer (2 M Tris-HCl, pH 7.4), and 100 μL of this mixture was added to each sample. The plate was monitored at 405 nm (with 490-nm background subtraction) for 40 min at 37°C by using a Bio-Tek ELx808 automated microplate reader (Bio-Tek Instruments, Inc., Winooski, VT, USA), equipped with KC4 software (Bio-Tek Instruments, Inc., Winooski, VT). The mean rate of optical density change was determined for each well, and the glucan concentration was determined by comparison to a standard curve. When absorbance was outside the range of the standard curve, the plasma or CSF samples were serially diluted in reagent-grade water and tested again.

### 2.7. Statistical Analysis

Comparisons between the groups were performed by using the Kruskal–Wallis test (nonparametric analysis of variance [ANOVA]) or Mann–Whitney U-test, as appropriate. A two-tailed *p* value of ≤ 0.05 was considered to be statistically significant. Values are expressed as means and standard error of the means (SEMs).

## 3. Results

### 3.1. Inoculum–Response Studies

**Figure 1:**
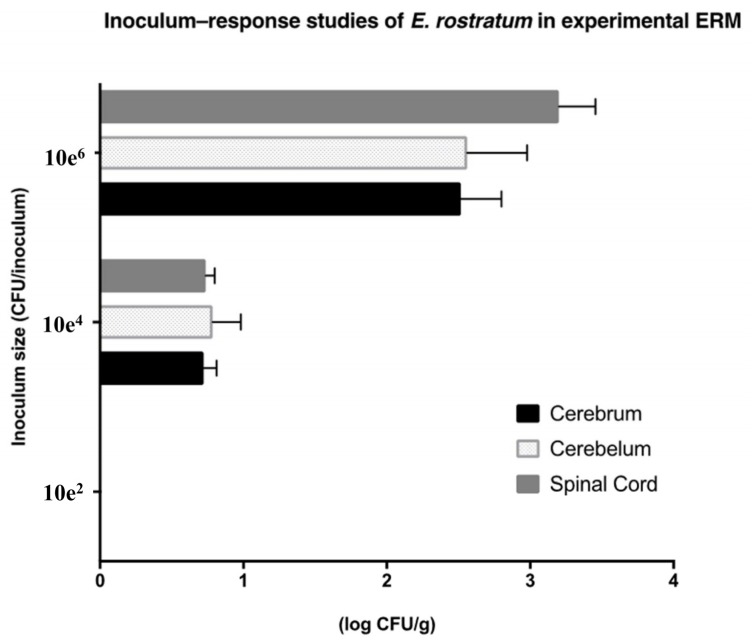
Inoculum–response studies in experimental *E. rostratum* meningoencephalitis (ERM), as measured in mean log CFU/g ± SEMs in cerebrum, cerebellum, and spinal cord versus log CFU/inoculum of organism in the immunocompromised rabbit model. Four rabbits were inoculated in each inoculum group. Values are expressed as mean log CFU/g ± SEMs.

There was a direct relationship between intracisternal inoculum size and tissue burden of *E. rostratum* (measured as log CFU/g ± SEMs) in the cerebrum, cerebellum, and spinal cord in experimental meningoencephalitis (Figure 1). An intracisternal inoculum of 1.0 × 10^2^ produced no detectible infection, while one of 1.0 × 10^4^ created a tissue burden of approximately 1.0 × 10^1^ CFU/g. By comparison, an inoculum size of 1.0 × 10^6^ established a reproducible infection with a tissue burden of approximately 1.0 × 10^3^ CFU/g.

### 3.2. Antifungal Therapy

**Figure 2:**
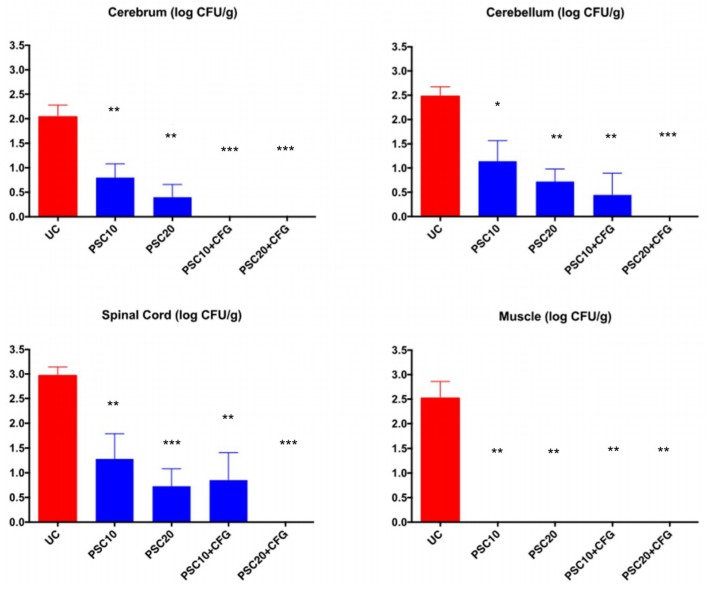
Response of *Exserohilum rostratum* meningitis in persistently neutropenic rabbits to antifungal therapy measured by mean residual tissue fungal burden (log CFU/g) in cerebrum, cerebellum, spinal cord, and paravertebral muscle. There were six rabbits in each treatment group and untreated control rabbits. Values are given as means of log CFU/g± SEMs. *, *p* < 0.05; **, *p* < 0.01, ***, *p* < 0.001, significant decrease in residual fungal burden in rabbits treated with posaconazole at 10 mg/kg/d, 20 mg/kg/d, and in combination with caspofungin in comparison to that of untreated controls (UC).

The response of *E. rostratum* meningoencephalitis in persistently neutropenic rabbits to antifungal therapy, as measured by mean residual tissue fungal burden (log CFU/g) in cerebrum, cerebellum, spinal cord, and paravertebral muscle is depicted in Figure 2. Posaconazole produced a significant reduction of residual fungal burden of *E. rostratum* in cerebrum, cerebellum, spinal cord, and paravertebral muscle (*p* < 0.01) in comparison to UC. The combination of PSC10+CFG and PSC20+CFG achieved full clearance of residual fungal burden from cerebrum, while only PSC20+CFG treated rabbits demonstrated clearance from cerebellum, spinal cord, and paravertebral muscle (*p* < 0.01). 

**Figure 3:**
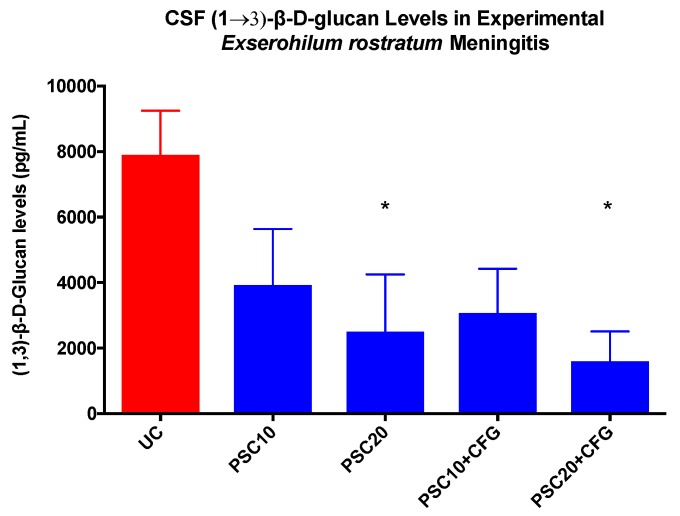
CSF (1→3)-β-d-glucan levels in rabbits of the experimental *Exserohilum rostratum* meningitis model in groups of untreated controls (UC), and rabbits receiving oral dose of posaconazole alone and in combination with caspofungin. There were six rabbits in each treatment group and untreated control rabbits. Values are given as mean (1→3)-β-d-glucan levels pg/mL ± SEMs; * *p* < 0.05.

CSF (1→3)-β-d-glucan levels in rabbits with experimental *E. rostratum* meningoencephalitis in untreated controls and rabbits receiving oral doses of posaconazole alone and in combination with caspofungin are depicted in Figure 3. There was a significant reduction of CSF (1→3)-β-d-glucan levels in rabbits treated with PSC20 and PSC20+CFG, in comparison to those of UC (*p* < 0.05), which paralleled the reductions observed in the residual fungal burden. 

## 4. Discussion

This study found that posaconazole alone or in combination with caspofungin demonstrated significant antifungal efficacy in the treatment of experimental *E.* rostratum meningoencephalitis in persistently neutropenic immunocompromised rabbits. Posaconazole significantly reduced the residual fungal burden of *E. rostratum* in the cerebrum, cerebellum, spinal cord, and paravertebral muscle. The combination of posaconazole plus caspofungin achieved full clearance of residual fungal burden from all tissues sampled. In parallel with these data, CSF (1→3)-β-d-glucan levels decreased significantly in rabbits treated with posaconazole, with or without caspofungin. These findings provide a preclinical foundation for the use of the combination of posaconazole with caspofungin in treatment of CNS phaeohyphomycosis. 

*Exserohilum rostratum* was studied in these experiments as a model pathogen of CNS phaeohyphomycosis. *Exserohilum rostratum* is a thermophilic dematiaceous mold, which was found to be an aggressive fungal pathogen in causing human disease in the recent multistate outbreak in the US [7,8]. Contamination of a methylprednisolone injection preparation by this pathogen resulted in more than 284 cases of *Exserohilum rostratum* meningitis. More than 20 of these cases of meningitis were fatal and many patients suffered severe morbidity [19,20,21]. This historically unprecedented public health threat underscored the importance of having pharmacodynamically sound therapies for mold infections of the CNS, especially those caused by dematiaceous molds, including *Exserohilum rostratum*, *Cladophialophora bantiana*, *Exophiala dermatitidis, and Ochroconis gallopava* [1,2,3,4,5,6,7,8,9].

In response to the severe morbidity and tragic mortality of dematiaceous mold infections of the central nervous system, we endeavored to investigate the activity of posaconazole and caspofungin in treatment of experimental *Exserohilum rostratum* meningoencephalitis as a model for CNS phaeohyphomycosis.

Currently, voriconazole or posaconazole are recommended as single agents for the treatment of CNS phaeohyphomycosis, based upon MICs, a paucity of preclinical laboratory animal studies, case reports, and small case series [9]. While patients with *Exserohilum rostratum* meningitis during the multistate outbreak were initially treated with a combination of liposomal amphotericin B and high dose voriconazole, this regimen proved to have unacceptable dose-limiting toxicity. In developing a broader strategy of treatment of CNS phaeohyphomycosis, for organisms with high MICs of 1 to 2 µg/mL, sustained voriconazole CSF concentrations may be difficult to achieve without reaching toxic plasma concentrations. The reduction of voriconazole dosage may then lead to progression of infection. Given the high mortality and incapacitating morbidity of dematiaceous molds, better therapy is critically needed. 

We therefore investigated the antifungal activity of posaconazole alone and in combination with caspofungin for treatment of these devastating infections. Such data are critical to arriving at informed decisions of for patient care and for the optimal design of clinical trials. The combination of posaconazole plus caspofungin exerted the greatest antifungal activity in eradicating organisms from the CNS tissues. 

This therapeutic response of the microbiological reduction of cerebral, cerebellar, spinal cord, and paravertebral muscle tissue was also reflected in parallel declines in CSF (1→3)-β-d-glucan levels. Initially reported in experimental hematogenous *Candida* meningoencephalitis (HCME) [22] and later demonstrated in pediatric patients with HCME [23], serial CSF (1→3)-β-d-glucan levels correlated strongly with the resolution of residual fungal burden in CNS tissue as well as the successful therapeutic outcome. Serial CSF (1→3)-β-d-glucan levels were also proven to be clinically predictive of the outcome in therapeutic monitoring of antifungal responses in treatment of *Exserohilum rostratum* meningitis [20]. By comparison, the quantitative PCR signal in patients with *Exserohilum rostratum* meningitis resolved after the initial CSF sample, further underscoring the value of serial CSF (1→3)-β-d-glucan levels for therapeutic monitoring [23]. Serial CSF (1→3)-β-d-glucan levels have been successfully used in the management of *Aspergillus* ventriculitis [24]. Given the presence of (1→3)-β-d-glucan in the cell walls of most pathogenic fungi of the Ascomycetes, we recommend that its utility be further studied in patients with CNS phaeohyphomycosis as a biomarker of monitoring therapeutic response through serial CSF sampling. 

## 5. Conclusions

In summary, this study demonstrates that posaconazole with or without caspofungin warrants further study in the treatment of patients with CNS phaeohyphomycosis. The addition of caspofungin to posaconazole may also increase the probability of successful treatment.
